# Angiotensin-converting enzyme inhibitors or angiotensin receptor blocker monotherapy retard deterioration of renal function in Taiwanese chronic kidney disease population

**DOI:** 10.1038/s41598-019-38991-z

**Published:** 2019-02-25

**Authors:** Cai-Mei Zheng, Jia-Yi Wang, Tzu-Ting Chen, Yun-Chun Wu, Yi-Lien Wu, Hsin-Ting Lin, Sheng-Po Chiu, Tian-Jong Chang, Jing-Quan Zheng, Nain-Feng Chu, Yu-Me Lin, Sui-Lung Su, Kuo-Cheng Lu, Jin-Shuen Chen, Fung-Chang Sung, Chien-Te Lee, Yu Yang, Shang-Jyh Hwang, Ming-Cheng Wang, Yung-Ho Hsu, Hung-Yi Chiou, Senyeong Kao, Mei-Yi Wu, Yuh-Feng Lin

**Affiliations:** 1 Graduate Institute of Clinical Medicine, College of Medicine, Taipei Medical University, Taipei, Taiwan; 2 Department of Internal Medicine, School of Medicine, College of Medicine, Taipei Medical University, Taipei, Taiwan; 3 Division of Nephrology, Department of Internal Medicine, Shuang Ho Hospital, Taipei Medical University, Taipei, Taiwan; 4 Graduate Institute of Medical Sciences, College of Medicine, Taipei Medical University, Taipei, Taiwan; 5 Department of Physiology, College of Medicine, Taipei Medical University, Taipei, Taiwan; 6 Institute of Epidemiology and Preventive Medicine, College of Public Health, National Taiwan University, Taipei, Taiwan; 7 Department of Primary Care Medicine, Shuang Ho Hospital, Taipei Medical University, Taipei, Taiwan; 8 School of Nursing, College of Nursing, Taipei Medical University, Taipei, Taiwan; 9 Kidney Disease Prevention Foundation, Taipei, Taiwan; 10 Department of Ophthalmology, Tri-Service General Hospital, National Defense Medical Center, Taipei, Taiwan; 11 Division of Endocrinology and Metabolism, Department of Internal Medicine, Tri-Service General Hospital Songshan Branch, National Defense Medical Center, Taipei, Taiwan; 12 Graduate Institute of Life Sciences, National Defense Medical Center, Taipei, Taiwan; 13 Performance Appraisal Section, Secretary Office, Shuang Ho Hospital, Taipei Medical University, Taipei, Taiwan; 14 Department of Critical Care Medicine, Shuang Ho Hospital, Taipei Medical University, Taipei, Taiwan; 15 School of Public Health, National Defense Medical Center, Taipei, Taiwan; 16 Department of Medical Education and Research, Kaohsiung Veterans General Hospital, Kaohsiung, Taiwan; 17 School of Public Health, College of Public Health and Nutrition, Taipei Medical University, Taipei, Taiwan; 18 Division of Nephrology, Department of Medicine, Fu-Jen Catholic University Hospital, School of Medicine, Fu-Jen Catholic University, Taipei, Taiwan; 19 Division of Nephrology, Department of Medicine, Tri-Service General Hospital, National Defense Medical Center, Taipei, Taiwan; 20 School of Public Health, Graduate Institute of Clinical Medical Science, China Medical University, Taichung, Taiwan; 21 Division of Nephrology, Kaohsiung Chang Gung Memorial Hospital, Chang Gung Medical University, Kaohsiung, Taiwan; 22 The Division of Nephrology, Changhua Christian Hospital, Changhua, Taiwan; 23 Division of Nephrology, Department of Medicine, Kaohsiung Medical University Hospital, Kaohsiung, Taiwan; 24 Division of Nephrology, Department of Internal Medicine, Cheng Kung University Medical Center, Tainan, Taiwan

## Abstract

It remains unclear how different uses of angiotensin-converting inhibitors (ACEIs) or angiotensin receptor blockers (ARBs) influence the progression of chronic kidney disease (CKD). This study explored CKD progression in a multicentre, longitudinal cohort study that included 2639 patients with CKD stage 1–5 and hypertension. Patients treated with ACEI or ARB for ≥90 days during a 6-mo period comprised the study group, or no treatment, comprised the control group. The study group was subdivided on the basis of treatment: ACEI monotherapy or ARB monotherapy. Progression of renal deterioration was defined by an average eGFR decline of more than 5 mL/min/1.73 m^2^/yr or the commencement of dialysis. With at least 1-year follow up, a progression of renal deterioration was demonstrated in 29.70% of the control group and 25.09% of the study group. Patients in the study group had significantly reduced progression of CKD with adjusted odds ratio 0.79 (95% confidence interval: 0.63–0.99). However, when ACEI monotherapy and ARB monotherapy were analyzed separately, none of their associations with CKD progression was statistically significant. In conclusion, ACEI or ARB monotherapy may retard the deterioration of renal function among patients with CKD and hypertension.

## Introduction

Chronic kidney disease (CKD) is a highly prevalent and concerning public health issue in the Taiwanese population^1,2^. Patients with CKD generally exhibit progressive deterioration in kidney function that concludes with end-stage renal disease (ESRD). Identifying effective measures to prevent and retard its progression is challenging but necessary^3,4^. For most types of renal diseases, effectively controlling blood pressure and minimizing proteinuria significantly attenuate kidney function deterioration. The MDRD Study 5 discovered that a reduction of proteinuria independently slowed the rate of GFR decline and that the renoprotective effect from lowering blood pressure depended on the level of proteinuria. Among antihypertensive agents, both angiotensin-converting enzyme (ACE) inhibitors (ACEI) and angiotensin II receptor blockers (ARBs) demonstrated a renoprotective effect attributable to both antihypertensive and antiproteinuric effects^5–7^. Further, these drugs interrupt the renal–angiotensin–aldosterone system (RAS)^8–11^, which plays a critical role in renal disease progression. Many clinical trials have demonstrated the value of ACEIs or ARBs for both patients with diabetes^10,12^ and those without^13^. Theoretically, the combination of an ACEI and an ARB might achieve a more complete inhibition of the RAS, and thereby achieve a stronger renoprotective effect. However, most published clinical trials and meta-analyses on combination therapy for renal protection have been inconclusive. A meta-analysis by Kunz *et al*. that examined 49 randomized trials (6181 patients) concluded that the combination of ACEIs and ARBs more effectively reduced proteinuria; however, most of the studies examined were small and did not provide details concerning adverse drug reactions^14^. Two recent clinical trials^15–17^ identified a decrease of albuminuria as a result of combination therapy with ACEIs and ARBs, but without slowing long-term renal deterioration. More adverse events, including acute kidney injury and hyperkalaemia, were associated with combination therapy^15–17^. We defined the progression of renal deterioration by an average eGFR decline of more than 5 mL/min/1.73 m^2^/yr or the commencement of dialysis. Given the uncertainties concerning the efficacy of ACEI or ARB treatment to slow the rapid progression of renal function, we conducted a study on a large multi-center cohort comprised of a Taiwanese population using the National Health Insurance Database in Taiwan, and examined the influence of ACEI monotherapy or ARB monotherapy on renal disease progression among patients with CKD and hypertension.

## Results

### Demographic characteristics of the patients

**Figure 1:**
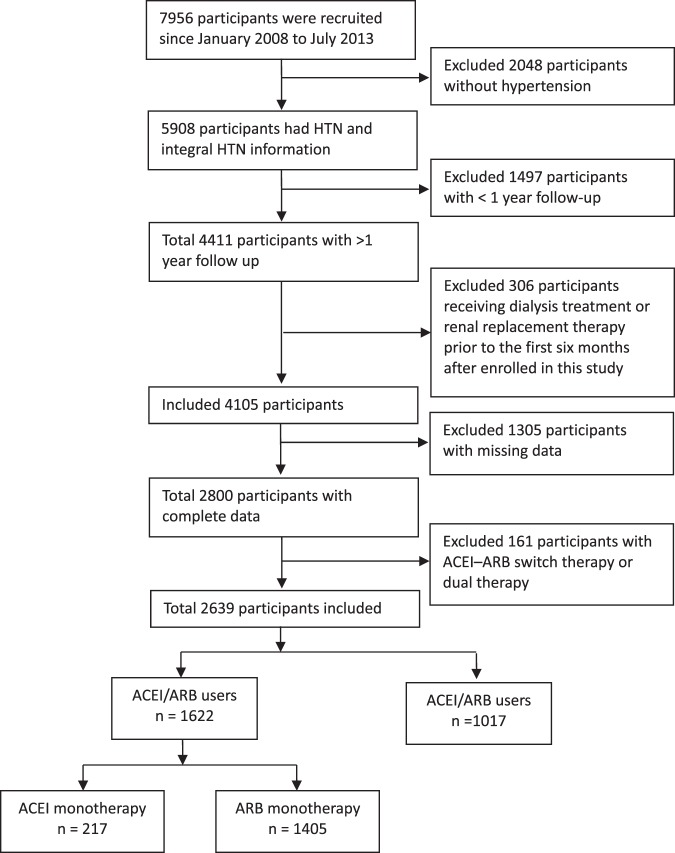
Flow chart of Patients Selection.

After excluding patients without hypertension, with less than 1-year follow up, receiving dialysis or renal transplant before enrolment, receiving dialysis or renal transplant within the first six months of observational period, with missing risk factor data, 2639 patients with CKD and hypertension were enrolled in this study (Fig. 1). We included 217 participants, 1405 participants, and 1017 participants in the ACEI monotherapy group, the ARB monotherapy group, and the control group, respectively. Among these patients, 1217 had early-stage CKD (CKD stage 1, stage 2, and stage 3a) and 1422 had advanced CKD (CKD stage 3b, stage 4, and stage 5). The mean age was 64.08 ± 13.17 and 66.99 ± 12.51 years in the study group and control group, respectively. There were more men than women in each group. The characteristics of these patients with CKD and hypertension are shown in Table 1. The control group tended to be older, to be more likely with previous diabetes mellitus (DM), to have a lower baseline eGFR, waist, BMI, serum K, Hb, and Hct, to have higher baseline triglyceride and serum phosphate level, and to be less likely to treat with an ACEI or ARB within 1 year prior to the index date compared with the study group.

**Table 1:** Baseline Characteristics of Patients with CKD Stages 1–5 and Hypertension.

Characteristic	ACEI mono-therapy (n = 217)	ARB mono-therapy (n = 1405)	Nonuser (n = 1017)	p-value
Age, mean (SD), y	63.31 ± 13.21	64.20 ± 13.17	66.99 ± 12.51	<0.0001
Age group, y				<0.0001
20–44	19 (8.76)	114 (8.11)	51 (5.01)	
45–64	87 (40.09)	568 (40.43)	354 (34.81)	
65–74	71 (32.72)	389 (27.69)	313 (30.78)	
≥75	40 (18.43)	334 (23.77)	299 (29.40)	
Male	127 (58.53)	838 (59.64)	592 (58.21)	0.7698
Comorbid conditions before the date index				
DM	94 (43.32)	706 (50.25)	443 (43.56)	0.0025
CAD	7 (3.23)	49 (3.49)	25 (2.46)	0.3464
Stroke	34 (15.67)	284 (20.21)	222 (21.83)	0.1174
Cancer	19 (8.76)	134 (9.54)	107 (10.52)	0.6179
Charlson comorbidity index				0.0003
≤3	139 (64.06)	680 (48.40)	493 (48.48)	
4–5	49 (22.58)	398 (28.33)	296 (29.11)	
>5	29 (13.36)	327 (23.27)	228 (22.42)	
Mean (SD)	3.15 ± 2.20	3.97 ± 2.38	3.95 ± 2.41	<0.0001
Antihypertensives used within 1 year before the index date				
ACEI	204 (94.01)	102 (7.26)	113 (11.11)	<0.0001
ARB	19 (8.76)	1332 (94.80)	365 (35.89)	<0.0001
α-Blockers	34 (15.67)	217 (15.44)	164 (16.13)	0.9017
β-Blockers	83 (38.25)	538 (38.29)	419 (41.20)	0.3292
Calcium channel blockers				
Nondihydropyridine	12 (5.53)	138 (9.82)	85 (8.36)	0.0872
Dihydropyridine	119 (54.84)	824 (58.65)	567 (55.75)	0.2770
Diuretics				
Loop diuretics	48 (22.12)	304 (21.64)	209 (20.55)	0.7707
Potassium sparing	8 (3.69)	94 (6.69)	58 (5.70)	0.1869
Other antihypertensives	9 (4.15)	40 (2.85)	32 (3.15)	0.5765
Baseline CKD stage				<0.0001
1	37 (17.05)	147 (10.46)	112 (11.01)	
2	63 (29.03)	306 (21.78)	196 (19.27)	
3A	34 (15.67)	199 (14.16)	123 (12.09)	
3B	38 (17.51)	258 (18.36)	166 (16.32)	
4	29 (13.36)	322 (22.92)	219 (21.53)	
5	16 (7.37)	173 (12.31)	201 (19.76)	
Smoking	55 (25.35)	367 (26.12)	277 (27.24)	0.7651
Alcohol	22 (10.14)	144 (10.25)	114 (11.21)	0.7301
Betel nut	14 (6.45)	84 (5.98)	61 (6.00)	0.9625
Exercise	77 (35.48)	478 (34.02)	353 (34.71)	0.8933
Waist, cm	86.73 ± 10.22	89.23 ± 11.58	87.41 ± 11.06	0.0001
Body mass index, kg/m^2^				<0.0001
<18.5	5 (2.30)	20 (1.42)	22 (2.16)	
18.5–24.9	99 (45.62)	595 (42.35)	533 (52.41)	
25–29.9	88 (40.55)	574 (40.85)	348 (34.22)	
≥30	25 (11.52)	216 (15.37)	114 (11.21)	
Fasting glucose, mg/dL	119.14 ± 42.24	118.37 ± 43.83	116.81 ± 41.61	0.7384
HbA1c, %	6.96 ± 1.36	6.97 ± 1.51	7.01 ± 2.71	0.2424
TG, mg/dL	138.29 ± 100.75	149.55 ± 105.23	137.65 ± 91.09	0.0007
Triglyceride, mg/dL	180.35 ± 39.59	180.47 ± 42.31	183.85 ± 42.35	0.1100
Serum Na	139.44 ± 3.70	139.35 ± 5.77	139.50 ± 7.60	0.9123
Serum K	4.47 ± 0.69	4.69 ± 4.80	4.55 ± 4.72	0.0038
Serum Ca	8.99 ± 0.84	9.13 ± 2.65	8.96 ± 0.69	0.0613
Serum P	3.77 ± 0.79	3.87 ± 1.35	4.01 ± 0.94	0.0010
Uric acid, mg/dL	6.80 ± 1.58	7.08 ± 2.24	7.12 ± 1.98	0.2482
Hb, mg/dL	12.53 ± 2.14	12.22 ± 2.37	11.98 ± 2.60	0.0025
Hct, mg/dL	37.19 ± 6.54	36.17 ± 6.03	35.29 ± 6.80	0.0004
Albumin, g/dL	4.08 ± 0.47	4.66 ± 15.17	4.07 ± 0.50	0.4343
UPCR	717.87 ± 1717.17	1092.31 ± 3387.21	1087.75 ± 2112.56	<0.0001
eGFR	58.56 ± 32.54	48.08 ± 31.04	45.40 ± 32.60	<0.0001

### Renal function deterioration events in patients treated with ACEIs and ARBs versus control groups

Table 2 shows the proportion of renal function deterioration events (eGFR decline events) among patients with CKD and hypertension. The number of eGFR decline events was 51 (23.5%), 356 (25.3%), and 302(29.7%) for the ACEI monotherapy group, the ARB monotherapy group, and the control group, respectively.

**Table 2:** Proportion of Events in Patients with CKD Stage 1–5 and Hypertension Comparing ACEI or ARB Users vs Nonusers.

Type of Treatment	No. of Events	No of Patients	Proportion (%)
ACEI/ARB users	407	1622	25.09
ACEI monotherapy	51	217	23.50
ARB monotherapy	356	1405	25.34
Nonusers	302	1017	29.70

We show the odds ratio (OR) of CKD progression in Table 3. When we compared the study group to the control group, the crude OR was 0.79 (95% confidence interval [CI]: 0.67–0.94). After adjusting for age, sex, previous comorbid conditions, and Charlson comorbidity index scores, previous ACEI or ARB use within 1 year before the index date, lifestyle characteristics, BMI, using immunosuppressants, the adjusted OR was 0.79 (95% CI: 0.63–0.99). When ACEI monotherapy and ARB monotherapy were analyzed separately, the adjusted ORs were 0.83 (95% CI: 0.49–1.41) and 0.85 (95% CI: 0.67–1.09) for ACEI monotherapy and ARB monotherapy, respectively.

**Table 3:** Study Outcomes: Risk in Patients with CKD Stages 1–5 and Hypertension; Comparing ACEI or ARB Users with Nonusers.

Type of Treatment	Study Outcome, OR (95% CI)			
	Unadjusted	*p*-value	Adjusted	*p*-value
ACEI/ARB user (n = 1622)	0.79 (0.67–0.94)	0.0095	0.79 (0.63–0.99)	0.0405
ACEI monotherapy (n = 217)	0.73 (0.52–1.02)	0.0677	0.83 (0.49–1.41)	0.4888
ARB monotherapy (n = 1405)	0.80 (0.67–0.96)	0.0174	0.85 (0.67–1.09)	0.2127
Nonuser (n = 1017)	1	—	1	—

Models were adjusted for age, sex, DM, CAD, CVA, cancer, Charlson score, Antihypertensives used within 1 year before the index date (10 items), smoking, alcohol consumption, betel nut chewing, UPCR and baseline eGFR, immunosuppressant.

OR, odds ratio; CI, confidence interval.

We further analyzed the beneficial effect of ACEI-ARB in CKD stage 1–3a and CKD stage 3b-5 as noted in Tables 4 and 5. The OR was less than one but without statistically significant among patients with CKD stage 1–3a (Table 4). In contrast, the adjusted OR was 0.73 (95% CI: 0.54–0.97) when we compared the study group to the control group among patients with CKD stage 3b-5 (Table 5). However, when ACEI monotherapy and ARB monotherapy were analyzed separately, none of their associations with CKD progression was statistically significant.

**Table 4:** Study Outcomes: Risk in Patients with CKD Stages 1–3a and Hypertension; Comparing ACEI or ARB Users with Nonusers.

Type of Treatment	Study Outcome, OR (95% CI)			
	Unadjusted	*p*-value	Adjusted	*p*-value
ACEI/ARB user (n = 786)	0.99 (0.76,1.30)	0.9516	0.89 (0.62,1.27)	0.5228
ACEI monotherapy (n = 134)	0.79 (0.50,1.25)	0.3109	0.68 (0.29,1.57)	0.3624
ARB monotherapy (n = 652)	1.04 (0.79,1.37)	0.7982	1.00 (0.67,1.50)	0.9928
Nonuser (n = 431)	1	—	1	—

Models were adjusted for age, sex, DM, CAD, CVA, cancer, Charlson score, Antihypertensives used within 1 year before the index date (10 items), smoking, alcohol consumption, betel nut chewing, UPCR, and immunosuppressant.

OR, odds ratio; CI, confidence interval.

**Table 5:** Study Outcomes: Risk in Patients with CKD Stages 3b–5 and Hypertension; Comparing ACEI or ARB Users with Nonusers.

Type of Treatment	Study Outcome, OR (95% CI)			
	Unadjusted	*p*-value	Adjusted	*p*-value
ACEI/ARB user (n = 836)	0.67 (0.53,0.85)	0.0009	0.73 (0.54,0.97)	0.0320
ACEI monotherapy (n = 83)	0.75 (0.45,1.26)	0.2795	0.91 (0.43,1.91)	0.7946
ARB monotherapy (n = 753)	0.66 (0.52,0.85)	0.0009	0.74 (0.54,1.02)	0.0647
Nonuser (n = 586)	1	—	1	—

Models were adjusted for age, sex, DM, CAD, CVA, cancer, Charlson score, Antihypertensives used within 1 year before the index date (10 items), smoking, alcohol consumption, betel nut chewing, UPCR, and immunosuppressant.

OR, odds ratio; CI, confidence interval.

Regarding patients with glomerulonephritis and immunosuppresants prescription, we analyzed the beneficial effect of study group and control group. We defined patients who had used immunosuppressants for more than one month within one year prior to the recruitment of this study as immunosuppressants user. The adjusted ORs were 0.55 (95% CI: 0.25,1.21) and 0.82 (95% CI: 0.64,1.03) among patients with and without immunosuppressants, respectively. We did not analyze ACEI monotherapy and ARB monotherapy separately because of small sample size.

### Risk factors for progression of renal deterioration with ACEI or ARB use

**Figure 2:**
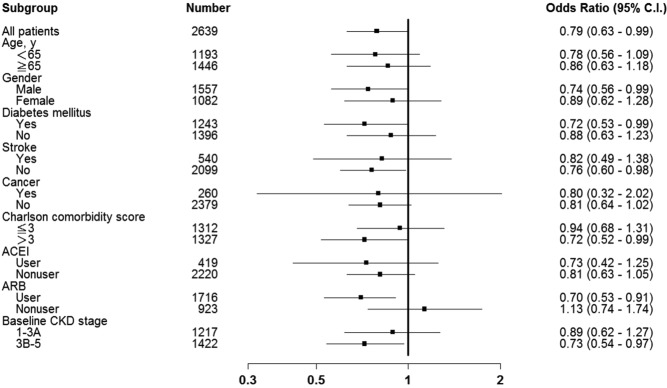
Adjusted Odds Ratios of Study Outcomes in Each Subgroup including age, sex, DM, stroke, cancer, Charlson comorbidity score, ACEI, ARB, CKD stage.

Figure 2 shows the subgroup analysis. After adjusting for several potential confounders, the ORs were lower than one and statistically significant in all patients (OR, 0.79; 95% CI, 0.63–0.99), male (OR, 0.74; 95% CI, 0.56–0.99), those with DM (OR, 0.72; 95% CI, 0.53–0.99), those without stroke (OR, 0.76; 95% CI, 0.60–0.98), those with Charlson comorbidity index >3 (OR, 0.72; 95% CI, 0.52–0.99), those with previous ARB use (OR, 0.70; 95% CI, 0.53–0.91), and those with advanced CKD (OR, 0.73; 95% CI, 0.54–0.97).

## Discussion

In this prospective cohort study, we investigated the relationship between ACEI or ARB therapy and the risk of eGFR decline in patients with CKD and hypertension. The multi-center cohort study enrolled patients with different stages of CKD to compare the influence of ACEI or ARB therapy and linked to the National Health Insurance database with corresponding data^18^. In contrast to definitions used in previous studies, we identified CKD progression events as either an annual average eGFR decline >5 mL/min/1.73 m^2^ or advancement to the dialysis stage.

At baseline, our control group was older than the study group, and had other comorbid conditions including CAD, stroke, and cancer; the Charlson comorbidity index scores were not significantly different. Significantly more patients in the study group had DM and more ACEI or ARB use within the year before the index date compared with the control group (Table 1). Unexpectedly, significantly later-stage CKD was exhibited in our control group compared with the study group, and this might explain the lesser use of ACEI and ARB within the year before the index date. Although we did not find a significant association between lifestyle characteristics in the two groups, the study group patients tended to be more obese than the control group. A comparison of different biochemical parameters revealed that the control group had less eGFR, more UPCR, lower haemoglobin (Hb) and haematocrit (Hct), and more serum phosphate (P) than the study group (Table 1).

Both ACEIs or ARBs have been noted to have antihypertensive and antiproteinuria effects because of different RAS-inhibition mechanisms^19–21^, and whether to use ACEIs or ARBs clinically in patients with advanced CKD remains a debatable topic^22^. Studies have detected the Ang-II escape phenomenon^19,23,24^ and poor local Ang-II inhibition with ACEI monotherapy^19^.

Consistent with other studies, the number of eGFR decline >5 mL/min/1.73 m^2^ or advancement to the dialysis stage events was significantly lower among ACEI or ARB users than in the nonuser group (29.7% in the nonuser group vs. 25.09% in the user group) (Table 2). A meta-analysis of 354 randomized controlled trials revealed that ACEIs or ARBs achieved comparable blood pressure (BP) reduction compared with thiazides, β blockers, and calcium channel blockers^25^. Matchar *et al*. determined that ACEIs and ARBs had similar long-term effects on BP^22^. However, several studies in diabetic nephropathy patients^26–28^ indicated that ACEIs and ARBs retard renal function deterioration through an antiproteinuric effect that goes beyond the pressure lowering effects. These studies confirmed that proteinuria at baseline and residual proteinuria 6–12 months after treatment predict long-term renal outcomes. Also, in nondiabetic patients with CKD and hypertension^29,30^, both ACEI and ARB slowed the progression of CKD through effects other than BP control. In the Ramipril Efficacy in Nephropathy (REIN) trial, ramipril retarded eGFR decline and the risk of end-stage kidney disease in patients with CKD with proteinuria of >3 g/day^31^. In the REIN-2 trial, no additional benefit was demonstrated from further BP reduction^32^. Further, Jafar *et al*. determined that the antiproteinuric effects of ACEIs are greater in patients with a high baseline urine protein excretion^33^. All of these studies indicated that ACEIs and ARBs are renoprotective independent of their antihypertensive effects.

We further analyzed the beneficial effects of ACEI or ARB in CKD stage 1–3a and CKD stage 3b-5 as noted in Tables 4 and 5. The results revealed no significant beneficial effects between users and nonusers in patients with CKD stage1–3a (Table 4) while significant protective effect in ACEI or ARB user group as compared to nonuser group in patients with CKD stage 3b-5 (Table 5). These data further demonstrated our unique finding that the beneficial effects of ACEI or ARB therapy did exist in patients with CKD stage 3b-5.

We concluded that ACEI or ARB monotherapy is associated with a lower proportion of eGFR decline events compared with the nonuser group (23.5%, 25.34%, 29.7% in ACEI monotherapy, ARB monotherapy, and non-user group, respectively. (Table 2). The risk of renal deterioration in the ACEI or ARB user group was significantly lower compared to non-users (Table 3). After adjusting for age, sex, previous comorbid conditions, Charlson comorbidity index scores, previous ACEI or ARB use within 1 year before the index date, lifestyle characteristics, BMI, baseline eGFR, and baseline UPCR, we also discerned a significant reduction in risk among patients in the ACEI or ARB monotherapy groups compared to non-user group.

Dual blockade of the RAS system with an ACEI and ARB have been frequently used clinically to prevent kidney disease^34^. In a meta-analysis of 14 randomized trials, Doulton *et al*.^35^ demonstrated that combination therapy caused an additional albeit small drop in BP and proteinuria compared with monotherapy. However, most of the included studies were relatively small trials with short-term follow up^35^. MacKinnon *et al*.^36^ determined that combination therapy caused a significant decline in proteinuria among diabetic and nondiabetic patients. They also discerned significantly high potassium levels with an nonsignificant decline in eGFR using combination therapy^36^. Other studies^14,37,38^ have also concluded that combination therapy significantly reduced proteinuria and hyperkalaemia levels and reduced or had no effect on eGFR levels. Recent clinical trials^15,17^ also demonstrated that an ACEI combined with an ARB results in more occurrences of hyperkalaemia and seriously adverse renal events, including acute kidney injury. Whether the use of ACEI and ARB should be individualized must be explored in future studies.

Using stratified analysis, we determined that the use of ACEI or ARB significantly retarded renal function deterioration consistently across most subgroups. The exceptions were including male patients, patients with DM, stroke, Charlson comorbidity index >3, previous ARB use within 1 year, and a baseline of advanced CKD. However, significant protection was exhibited among patients with late-stage CKD. Our finding is consistent with Hsu *et al*.^39^, who determined that the use of ACEI or ARB reduced the risks of both dialysis and a composite of dialysis and death in a median follow-up period of 7 months. We contend that a definite protective effect exists from the use of ACEI or ARB, especially among those with advanced CKD, and recommend their use in all levels of CKD with close monitoring for adverse events.

This national cohort study is to provide empirical evidence demonstrating the effects of uses of ACEI or ARB medication for lowering eGFR. This study had several advantages. First, we examined a large national multi-center research with patients from a comprehensive nationwide database. Second, information on demographic characteristics and health-related behaviours were collected through face-to-face interviews conducted by well-trained interviewers to ensure data quality. Third, we linked two large data sources (the Epidemiology and Risk Factors Surveillance of CKD database and the National Health Insurance database) to include biochemical data when analysing the results to ensure the quality of study outcomes. Detailed biochemistry data was available to define the stage and severity of CKD.

However, some limitations were encountered when conducting this study. First, because patients voluntarily enrolled in the study, a potential selection bias was unavoidable. Second, variables of clinical disease were collected using a structured questionnaire, introducing the potential for underestimation of certain test results. Third, this study did not contain drug-use details concerning dosage, which might influence data analysis. In conclusion, this study determined the influence of ACEI or ARB uses on progression of renal deteriorationa mong patients at various stages of CKD and hypertension. We determined that ACEI or ARB use significantly retarded renal function deterioration through all stages of CKD. Moreover, a significant renoprotective effect was noted with medication use in later CKD stages (eGFR ≤ 45 mL/min). Thus, ACEI or ARB monotherapy may considered in patients with CKD and hypertension and close monitored about side effects.

## Materials and Methods

### Ethics statement

This study was reviewed and approved by the institutional ethical committee of Taipei Medical University - Shuang Ho Hospital (TMU-JIRB 201204036), Tri-Service General Hospital (TSGHIRB100-05-197), Cardinal Tien Hospital (TMU-JIRB 201204035), Changhua Christian Hospital (CCHIRB 20405), Kaohsiung Medical University Chung-Ho Memorial Hospital (KMUHIRB 20120019), Kaohsiung Chang Gung Memorial Hospital (101–1096B), National Cheng Kung University Hospital (A-ER-101-117), and China Medical University Hospital (DMR101-IRB2-273(CR-1)). After a complete explanation of the study, written informed consent was obtained from all participants. All clinical and biological samples were collected after patient consent, and all experiments were performed in accordance with relevant guidelines and regulations.

### Study cohort

We conducted a multicentre, longitudinal cohort study based on the Epidemiology and Risk Factors Surveillance of CKD database from 2008 to 2013; the database is maintained separately by the Bureau of Health Promotion, Ministry of Health and Welfare, Taiwan. Epidemiology and Risk Factors Surveillance of CKD database is including 7956 patients with CKD and ages younger than 20 years old from 14 hospitals. The same medical laboratory criteria and protocol were used in our study hospitals, and the value of serum creatinine was derived from a different hospital, but could be compared and standardized. In addition, we linked the biochemical laboratory data to the health insurance database in Taiwan from 2004 to 2013. All registrations and claim data of participants were available to this study (i.e. age, sex, dates of clinical visits, diagnosis codes, prescriptions, surgeries, and expenditure of all treatments). In this study, patients were continually followed from the baseline date to the end of the study period (June 18, 2015), and patients were re-examined in the same hospital to control for individual variation. Patients who were without previous diagnose of hypertension, less than 1-year follow up, receiving dialysis treatment or renal replacement therapy before including in this study, receiving dialysis treatment or renal replacement therapy within the first six months after enrolled in this study, with missing data for BMI or UPCR, receiving ACEI–ARB switch therapy or dual therapy were excluded.

### Measurements and variable definitions

Patients were grouped according to the use of ACEI and ARB drugs during the first six months of observational period; if the patients were treated with ACEIs and ARBs for at least 90 days within a 6-mo period, they were categorized into the medication group, and the others were classified as the control group. The study group was subdivided according to the nature of treatment, such as ACEI monotherapy and ARB monotherapy. We defined that the ACEI monotherapy group used only ACEI for more than 90 days during the first six months of observational period, and we defined that the ARB monotherapy group used only ARB for more than 90 days during the first six months of observational period.

Renal deterioration progression was defined as an average eGFR decline of more than 5 mL/min/1.73 m^2^ per year or progression into the dialysis stage^40^. CKD was defined according to the Kidney Disease Outcomes Quality Initiative guidelines^41^, and was evaluated using eGFR, which was calculated using the Chronic Kidney Disease–Epidemiology Collaboration equation, as recommended by KDIGO guidelines. CKD was classified as follows: CKD stage 1, eGFR ≥90 mL/min/1.73 m^2^ and the presence of kidney damage (i.e., proteinuria dipsticks ≥1+, UPCR ≥150, or urine albumin-to-creatinine ratio [UACR] ≥30); CKD stage 2, eGFR = 60–89 mL/min/1.73 m^2^ and the presence of kidney damage (i.e., proteinuria dipsticks ≥1+, UPCR ≥150, or UACR ≥30); CKD stage 3a, eGFR = 45–59 mL/min/1.73 m^2^; CKD stage 3b, eGFR = 30–44 mL/min/1.73 m2; CKD stage 4, eGFR = 15–29 mL/min/1.73 m^2^; and CKD stage 5, eGFR <15 mL/min/1.73 m^2^ ^42^.

Baseline variables included demographic characteristics, namely age and sex; clinical variables were DM, CAD, stroke, and cancer; physical examination variables were waist circumference, BMI, systolic blood pressure (SBP), and diastolic blood pressure (DBP); laboratory test variables were levels of serum creatinine, blood urea nitrogen, uric acid, glycated haemoglobin (HbA1C), triglyceride, total cholesterol, and proteinuria; and health-related behaviours included cigarette smoking, alcohol consumption, betel nut chewing, and exercise. The demographic and health-related behaviour data were collected using a structured questionnaire. The physical examination and laboratory variables were obtained through medical chart reviews, and the clinical variables were obtained from the health insurance database. BMI was classified as <18.5, 18.5 to 24.9, and ≥25 kg/m^2^. Cigarette smoking was dichotomized as smoking (smoking ≥100 cigarettes during the patient’s lifetime) and never smoking. Alcohol consumption was dichotomized as current and noncurrent drinking.

### Statistical analysis

The characteristics of treatment groups and reference group were compared using the chi-squared test for categorical variables, ANOVA test for continuous variables with normal distribution, and Kruskal-Wallis test for continuous variables with non-normal distribution. We used logistic regression models, including all potential confounders, to evaluate the association between ACEI and ARB use and eGFR decline. We first estimated the crude ORs, and then we estimated the adjusted ORs by including age, sex, previous comorbid conditions, such as DM, stroke, and cancer, Charlson comorbidity index scores, use of ACEI and ARB medication within the previous 1 year, cigarette smoking, alcohol consumption, betel nut chewing, BMI, baseline UPCR, and baseline eGFR in the model. Next, we did several subgroup analyses stratified by age, sex, DM, Stroke, cancer, Charlson comorbidity index, baseline CKD stage, prevalence ACEI user, prevalence ARB user, and immunosuppressants prescription. All analyses and calculations were performed using SAS Version 9.4 (SAS Institute Inc, Cary, NC).

## Data Availability

The datasets generated during and/or analysed during the current study are not publicly available due to ethical policy but are available from the corresponding author on reasonable request.
