# Author Correction: Angiotensin-converting enzyme inhibitors or angiotensin receptor blocker monotherapy retard deterioration of renal function in Taiwanese chronic kidney disease population

**DOI:** 10.1038/s41598-020-63162-w

**Published:** 2020-04-15

**Authors:** Cai-Mei Zheng, Jia-Yi Wang, Tzu-Ting Chen, Yun-Chun Wu, Yi-Lien Wu, Hsin-Ting Lin, Sheng-Po Chiu, Tian-Jong Chang, Jing-Quan Zheng, Nain-Feng Chu, Yu-Me Lin, Sui-Lung Su, Kuo-Cheng Lu, Jin-Shuen Chen, Fung-Chang Sung, Chien-Te Lee, Yu Yang, Shang-Jyh Hwang, Ming-Cheng Wang, Yung-Ho Hsu, Hung-Yi Chiou, Senyeong Kao, Mei-Yi Wu, Yuh-Feng Lin

**Affiliations:** 1 Graduate Institute of Clinical Medicine, College of Medicine, Taipei Medical University, Taipei, Taiwan; 2 Division on Nephrology, Department of Internal Medicine, School of Medicine, College of Medicine, Taipei Medical University, Taipei, Taiwan; 3 Division of Nephrology, Department of Internal Medicine, Shuang Ho Hospital, Taipei Medical University, Taipei, Taiwan; 4 Graduate Institute of Medical Sciences, College of Medicine, Taipei Medical University, Taipei, Taiwan; 5 Department of Physiology, College of Medicine, Taipei Medical University, Taipei, Taiwan; 6 Institute of Epidemiology and Preventive Medicine, College of Public Health, National Taiwan University, Taipei, Taiwan; 7 Department of Primary Care Medicine, Shuang Ho Hospital, Taipei Medical University, Taipei, Taiwan; 8 School of Nursing, College of Nursing, Taipei Medical University, Taipei, Taiwan; 9 Kidney Disease Prevention Foundation, Taipei, Taiwan; 10 Department of Ophthalmology, Tri-Service General Hospital, National Defense Medical Center, Taipei, Taiwan; 11 Division of Endocrinology and Metabolism, Department of Internal Medicine, Tri-Service General Hospital Songshan Branch, National Defense Medical Center, Taipei, Taiwan; 12 Graduate Institute of Life Sciences, National Defense Medical Center, Taipei, Taiwan; 13 Performance Appraisal Section, Secretary Office, Shuang Ho Hospital, Taipei Medical University, Taipei, Taiwan; 14 Department of Critical Care Medicine, Shuang Ho Hospital, Taipei Medical University, Taipei, Taiwan; 15 School of Public Health, National Defense Medical Center, Taipei, Taiwan; 16 Department of Medical Education and Research, Kaohsiung Veterans General Hospital, Kaohsiung, Taiwan; 17 School of Public Health, College of Public Health and Nutrition, Taipei Medical University, Taipei, Taiwan; 18 Division of Nephrology, Department of Medicine, Fu-Jen Catholic University Hospital, School of Medicine, Fu-Jen Catholic University, Taipei, Taiwan; 19 Division of Nephrology, Department of Medicine, Tri-Service General Hospital, National Defense Medical Center, Taipei, Taiwan; 20 School of Public Health, Graduate Institute of Clinical Medical Science, China Medical University, Taichung, Taiwan; 21 Division of Nephrology, Kaohsiung Chang Gung Memorial Hospital, Chang Gung Medical University, Kaohsiung, Taiwan; 22 The Division of Nephrology, Changhua Christian Hospital, Changhua, Taiwan; 23 Division of Nephrology, Department of Medicine, Kaohsiung Medical University Hospital, Kaohsiung, Taiwan; 24 Division of Nephrology, Department of Internal Medicine, Cheng Kung University Medical Center, Tainan, Taiwan

Correction to: *Scientific Reports* 10.1038/s41598-019-38991-z, published online 25 February 2019

The original version of this Article contained an error in Affiliation 2, which was incorrectly given as ‘Department of Internal Medicine, School of Medicine, College of Medicine, Taipei Medical University, Taipei, Taiwan’. The correct affiliation is listed below:

Division on Nephrology, Department of Internal Medicine, School of Medicine, College of Medicine, Taipei Medical University, Taipei, Taiwan

In addition, in Figure 1 the bottom right panel was incorrectly labelled as ‘ACEI/ARB users’.

These errors have now been corrected in the HTML and PDF versions of the Article.

